# Mesenchymal stromal cells prevent progression of liver fibrosis in a novel zebrafish embryo model

**DOI:** 10.1038/s41598-018-34351-5

**Published:** 2018-10-30

**Authors:** Danny van der Helm, Arwin Groenewoud, Eveline S. M. de Jonge-Muller, Marieke. C. Barnhoorn, Mark J. A. Schoonderwoerd, Minneke J. Coenraad, Lukas J. A. C. Hawinkels, B. Ewa Snaar-Jagalska, Bart van Hoek, Hein W. Verspaget

**Affiliations:** 1 Department of Gastroenterology and Hepatology, Leiden University Medical Center, Leiden, The Netherlands; 2 Department of Animal Science and Health, Leiden University, Leiden, The Netherlands

## Abstract

Chronic liver damage leads to the onset of fibrogenesis. Rodent models for liver fibrosis have been widely used, but are less suitable for screening purposes. Therefore the aim of our study was to design a novel model for liver fibrosis in zebrafish embryos, suitable for high throughput screening. Furthermore, we evaluated the efficacy of mesenchymal stromal cells (MSCs) to inhibit the fibrotic process and thereby the applicability of this model to evaluate therapeutic responses. Zebrafish embryos were exposed to TAA or CCL4 and mRNA levels of fibrosis-related genes (Collagen-1α1, Hand-2, and Acta-2) and tissue damage-related genes (TGF-β and SDF-1a, SDF-1b) were determined, while Sirius-red staining was used to estimate collagen deposition. Three days after start of TAA exposure, MSCs were injected after which the fibrotic response was determined. In contrast to CCL4, TAA resulted in an upregulation of the fibrosis-related genes, increased extracellular matrix deposition and decreased liver sizes suggesting the onset of fibrosis. The applicability of this model to evaluate therapeutic responses was shown by local treatment with MSCs which resulted in decreased expression of the fibrosis-related RNA markers. In conclusion, TAA induces liver fibrosis in zebrafish embryos, thereby providing a promising model for future mechanistic and therapeutic studies.

## Introduction

The liver is a vital organ with distinct functions like detoxification, metabolism and immune defence. Chronic exposure of the liver to injurying circumstances, like viral hepatitis infection, chronic alcohol abuse, steatohepatitis and cholestatic disease results to apoptotic hepatocytes and subsequent stellate cell activation which differentiate into myofibroblasts^1–3^. These myofibroblasts are the main source of progressive deposition of extracellular matrix (ECM) components, which leads to fibrogenesis^1–3^.

To understand the pathogenesis and investigate novel therapeutic interventions diverse model systems for fibrogenesis have been used. These include *in vivo* mouse and rat models, mostly based on the well-known carbon tetrachloride (CCL4) or thioacetamide (TAA) induced liver fibrosis. Both compounds are metabolised by the hepatocytes into hepatotoxic metabolites leading to apoptosis of these cells and subsequently activation and proliferation of stellate cells^4,5^. These mouse models have been proven valuable, but are expensive and time consuming, as it takes 6 and 12 weeks to induce a chronic fibrosis or cirrhosis, respectively. Furthermore, administration of the toxic compounds in mice may cause acute toxicity, sometimes leading to death of the mice. Finally, using the CCL4 method non-liver related side effects like intraperitoneal adhesions have been reported^4^. These drawbacks make these models less attractive for high throughput compound screening.

Zebrafish embryos are often used to perform high throughput drug screens^6^. Beneficial characteristics of zebrafish embryos as model organisms include the convenience to house these small-sized animals, the short generation time, ease of embryo accessibility, low costs and transparency of the organism in the early development^7–9^. With respect to liver physiology, the zebrafish shows a 70% similarity to the human liver, including the same cell types as observed in the human liver (e.g., hepatocytes, stellate cells, biliary cells and endothelial cells)^7,10^. In mature zebrafish, TAA or ethanol dissolved in aquarium water has been reported to induce liver fibrosis with similar mechanism as observed in humans^11–13^. This makes zebrafish embryos an attractive model for liver fibrosis and to screen new therapeutic compounds in a high throughput screening fashion.

A limited number of studies have reported on models of liver fibrosis in zebrafish embryos. Addition of ethanol in aquarium water shows an acute fibrotic response in zebrafish embryos, characterised by increased collagen and Hand-2 (stellate cell proliferation marker) protein levels^14–17^. However, fibrotic effects of the hepatotoxic compounds CCL4 and TAA has not been investigated. In the present study we aim to translate the widely used CCL4 and TAA mouse models for liver fibrosis to zebrafish embryos in order to obtain a new model which is suitable for high throughput studies and show its applicability to study therapeutic effects.

Liver fibrosis is one of the most prevalent diseases in the western world and no real treatment for end-stage cirrhosis, besides liver transplantation, exists^18–20^. Novel treatments to reverse fibrogenesis are needed. Promising results regarding the effect on fibrogenesis have been obtained from *in vivo* experimental and clinical studies using mesenchymal stromal cells (MSCs)^21–25^. MSCs are stromal cells which can be easily isolated from various tissue sources, expanded in culture and are not rejected after transplantation^23,26,27^. Positive functional characteristics of MSCs are their ability to modulate the immune system and their role in the repair and regeneration of damaged tissue^23,28^. In relation to liver fibrosis several animal studies already showed that MSCs can inhibit and reverse the fibrotic process^24,25,29,30^. Supposed mechanisms for this effect include improvement of hepatocyte survival, inhibition of stellate cell activation, and proliferation and silencing of myofibroblasts^24,25,29,31,32^. MSCs and fibroblasts are both stromal cells with overlapping functions in the organisation of extracellular matrix. However, studies comparing both cell types side by side are limited. Therefore, we established a new high throughput zebrafish embryo model for liver fibrosis and evaluated its applicability to test potential new therapeutics by testing the ability of injected MSCs and fibroblasts to reduce the induction of liver fibrosis.

## Results

### CCL4 administration does not induce liver fibrosis in zebrafish embryos

**Figure 1:**
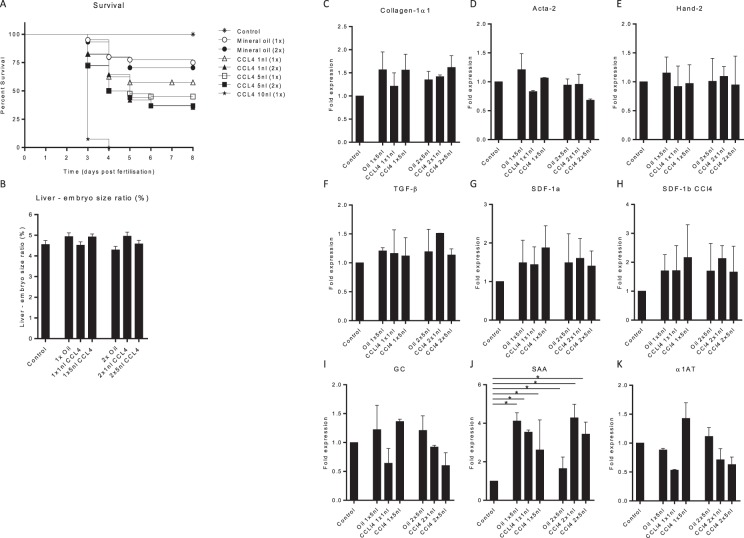
CCL4 administration does not induce liver fibrosis in de zebrafish embryos. Zebrafish embryos (2dpf) were injected in the yolk sac with various volumes and frequencies of 0.25M CCL4 or mineral oil as a control. (**A**) Survival of the embryos during CCL4 treatment (N = 50 embryos, ±SEM). (**B**) At 8dpf the embryos were imaged to measure the sizes of the liver and total embryo in order to calculate the liver to embryo size ratio (N = 50 embryos). (**C**–**K**) Quantitative PCR for mRNA expression of fibrotic, tissue damage and liver function related genes after CCL4 administration. Expression levels of Collagen1α1, Acta-2, Hand-2, TGFβ, SDF-1a, SDF1-b, GC, SAA and α1AT are normalized to RPP and to heathy control embryos. The graphs represent values of three independent experiments (n = 3, ±SEM). *p ≤ 0.05.

Due to the hydrophobic characteristic of CCL4, this compound could not be dissolved in the egg water and was therefore injected in the yolk sac of the zebrafish embryo. To find the optimal dose, 0.25 M CCL4 was 1 (2dpf) or 2 times (2 and 4dpf) injected to the yolk sac of the embryo. Survival analysis of the CCL4 treated embryos indicated toxic effects at higher volumes/doses. A single injection of 10 nl CCL4 did lead to phenotypic toxic effects (like oedema in heart cavity or malformations) and was lethal for all embryos within 2 days after administration. Embryos receiving two CCL4 injections resulted in 40–50% survival regardless of the injected volume (Fig. 1A). In these groups no phenotypic toxic effects were observed. Furthermore, CCL4 administration did not lead to differences in embryo and liver sizes compared to the control groups (Fig. 1B). When analysing extracellular matrix deposition by Sirius-red staining, no structures of collagen deposition were observed (data not shown). Furthermore expression of the fibrosis-related genes (collagen-1α1, Acta-2 and Hand-2) (Fig. 1C–E) was not affected. Also the inflammation and damage (TGF-β, SDF-1a, SDF-1b) or liver function (GC or α1AT) related mRNA expression levels were not consistently affected (Fig. 1F–K). Only the acute phase protein SAA was significantly upregulated in all groups compared to the control embryos (Fig. 1J). These data indicate an acute toxic effect of CCL4 injection without signs of induction of liver fibrosis.

### TAA treatment induces liver fibrosis in zebrafish embryos

**Figure 2:**
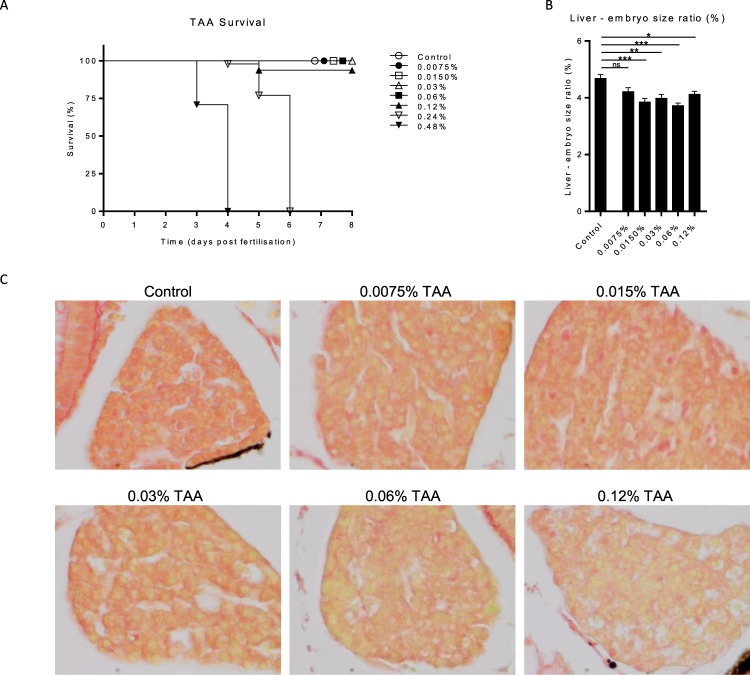
Thioacetamide titration in zebrafish embryos. Zebrafish embryos (2dpf) were treated until 8dpf with different concentrations of TAA in egg water. (**A**) Survival of the embryos during TAA treatment (N = 50 embryos). (**B**) At 8dpf the embryos were imaged to measure the sizes of the liver and total embryo in order to calculate the liver to embryo size ratio (N = 2, ±SEM). (**C**) Sirius-red stained section of TAA treated zebrafish embryo livers (8dpf, 400x magnification). *p ≤ **p ≤ 0.01, ***p ≤ 0.001.

**Figure 3:**
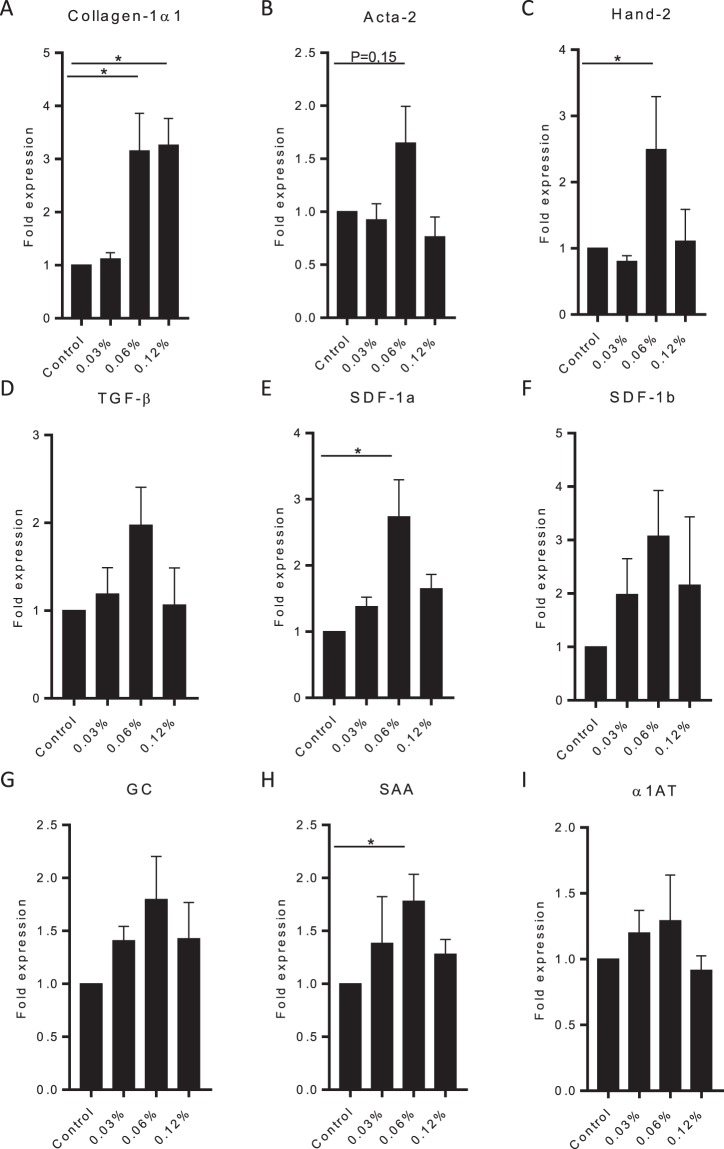
RNA expression levels after TAA treatment. Quantitative PCR for mRNA expression of fibrotic, tissue damage and liver function genes after TAA treatment (8dpf). (**A**–**I**) Expression levels of Collagen1α1, Acta-2, Hand-2, TGF-β, SDF-1a, SDF1-b, GC, SAA and α1AT are normalized to RPP and to heathy control embryos. The graphs represent values of three independent experiments (n = 3, ±SEM). *p ≤ 0.05.

**Figure 4:**
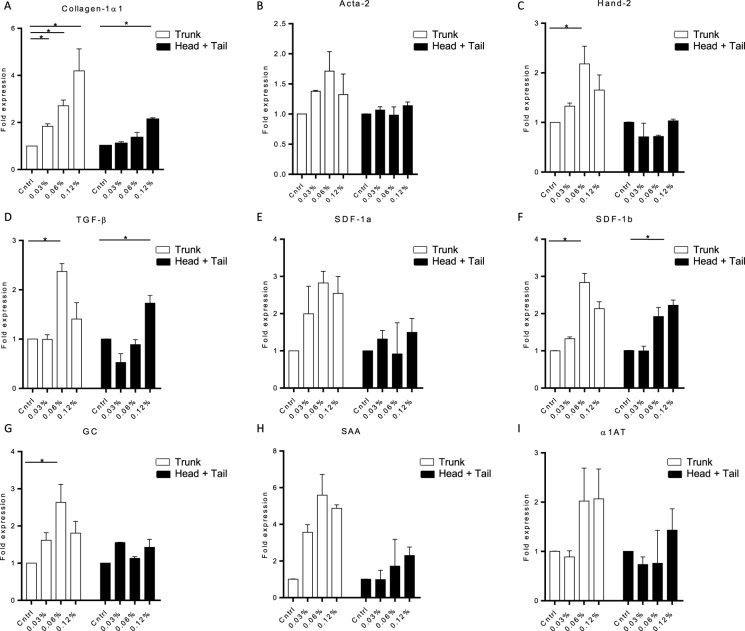
Gene expression changes in the trunk region compared to head and tail pools. Quantitative PCR for mRNA expression of fibrotic, tissue damage and liver function genes after TAA treatment. The trunk region was compared to het pooled head and tail RNA expression levels. (**A**–**I**) Expression levels of Collagen1α1, Acta-2, Hand-2, TGF-β, SDF-1a, SDF1-b, GC, SAA and α1AT are normalized to RPP and to heathy control embryos. The graphs represent values of three independent experiments (n = 3, ±SEM). *p ≤ 0.05.

TAA is a hydrophilic compound which can be easily dissolved in egg water. After analysing increasing doses of TAA, we observed that treatment with 0.24% and 0.48% TAA induced an acute toxic effect with oedema in the heart cavity and all embryos died within 2 to 4 days after start of the treatment (Fig. 2A). None of the other groups showed any signs of toxicity, also the total embryo size was not affected (data not shown). Liver size relative to total embryo, however, was smaller in all groups that received TAA, except for the lowest concentration indicating an TAA effect on the liver (Fig. 2B). To analyse collagen deposition a Sirius-red staining was performed. This analysis revealed subtle structures of collagen deposition between the hepatocytes in the 0.03%, 0.06% and 0.12% TAA treated embryos (Fig. 2C). Also the cells and total liver architecture seemed to be disturbed in the higher doses compared to control and lower dosages (0.0075% and 0.015% TAA). These results indicate an effect of TAA treatment on the liver and a possible onset of fibrogenesis in the 0.03%, 0.06% and 0.12% TAA treatment groups. Furthermore, qPCR analysis showed increased expression of collagen mRNA levels in the 0.06% and 0.12% TAA groups (Fig. 3A). Acta-2 and Hand-2 expression levels were higher in the 0.06% TAA treated group compared to the other groups (Fig. 3B,C). Furthermore, the tissue damage/inflammatory genes, TGF-β, SDF-1a, SDF-1b, show a trend toward higher expression levels in the 0.06% TAA treated group (Fig. 3D–F). The liver function related genes, GC and SAA showed a similar trend. No significant effect on α1AT RNA level was observed (Fig. 3G–I). To study these expression levels more locally, head, trunk (including the liver) and tail of the embryos were separated and RNA was isolated. Observed expression levels of fibrotic, inflammatory/damage and liver function related genes in the trunk reveal a similar trend as the expression levels found in whole embryo RNA isolations. Furthermore, this effect was not observed in pooled head and tail RNA samples of 0.06% TAA treated embryos (Fig. 4A–I). Altogether these data indicate an consistent induction of liver fibrosis in the 0.06% TAA treatment group, as shown by increased expression of fibrosis related genes and increased deposition of collagen.

### MSCs ameliorate TAA induced fibrosis in zebrafish embryos

To study if therapeutic interventions can be tested in this model, MSCs and fibroblast were used. First, the MSCs were phenotypical and functionally characterized. MSCs were positive for the membrane MSC markers CD29, CD44, CD105, CD106 and SCA-1 and negative for the hematopoietic marker CD45 and endothelial marker CD31 (Supplemental Fig. 2A). To analyze their multipotency differentiation assays followed by fast blue, alizarin-red and oil-red-o staining were performed. Positive staining for osteogenic markers, and presence of lipid droplets in the adipogenic differentiation medium showed that the MSCs can undergo osteogenic- and adipogenic differentiation (Supplemental Fig. 2B). Based on the expressed membrane markers and the ability to differentiate into different lineages these cells were defined as MSCs.

**Figure 5:**
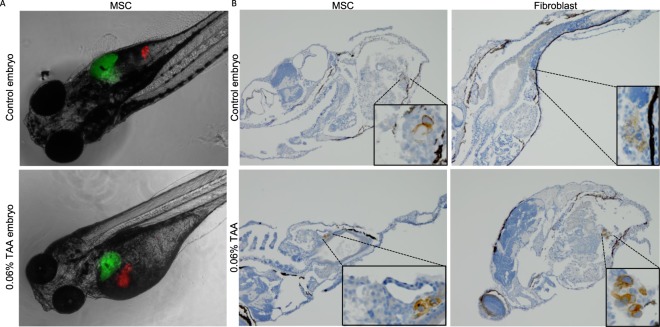
MSC and fibroblast tracing at 8dpf. During fibrotic induction (5dpf) with 0.06% TAA fibroblasts and MSCs were injected in close proximity to the liver. (**A**) Representative fluorescence images of zebrafish after MSC administration (8dpf) liver (green) and MSCs (red) (20x magnification). (**B**) Representative Vimentin stained section of fibroblast or MSC treated embryos (20x magnification).

MSCs, fibroblasts or a solvent control (PVP), were injected 3 days after the start of 0.06% TAA treatment (5dpf), in close proximity to the liver. MSCs expressing the RFP construct could be detected in 7 out of 8 of the embryos (87.5%) at the end of the experiment in the TAA treated group. Surprisingly, MSCs injected in control embryos could be traced in only 2 out of 8 embryos (25%). Traced MSCs were observed around the injection area and further cell migration to, for example, the tail, was not observed (Fig. 5A). In concordance, mouse specific Vimentin staining also revealed that both MSCs and fibroblasts could be traced until the end of the experiment (Fig. 5B).

**Figure 6:**
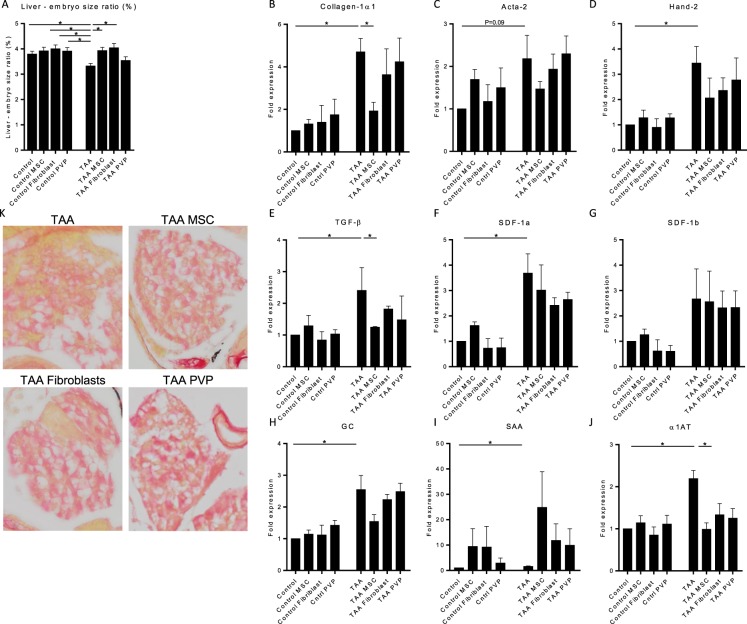
MSCs prevent the progression of liver fibrosis in zebrafish embryos. Quantitative PCR for mRNA expression of fibrotic, tissue damage and liver function genes after TAA treatment and MSC, Fibroblast or PVP injections. (**A**) At 8dpf the embryos were imaged to measure the sizes of the liver and total embryo in order to calculate the liver to embryo size ratio (N = 2, ±SEM). (**B**–**J**) Expression levels of Collagen1α1, Acta-2, Hand-2, TGF-β, SDF-1a, SDF1-b, GC, SAA and α1AT are normalized to RPP and to heathy control embryos. The graphs represent values of three independent experiments (n = 3, ±SEM). *p ≤ 0.05.

Treatment with either MSCs or fibroblasts both abolished the effect of TAA treatment on liver size (Fig. 6A), compared to control. MSC treatment in embryos with liver fibrosis consistently reduced the RNA expression levels of collagen and TGF-β compared to embryos without MSC treatment (Fig. 6B,E). Acta-2 and Hand-2 expression were also downregulated, although this did not reach statistical significance (Fig. 6C,D). Fibroblast or PVP solvent control administration resulted in an intermediate reduction of these genes. No changes in SDF-1a and SDF-1b expression levels were observed after MSC treatment. Expression levels of GC and α1AT upon MSC treatment revealed a trend towards normal levels (Fig. 6H,J). SAA expression levels were non-significantly elevated upon MSC administration (Fig. 6I). Sirius-red staining for collagen deposition revealed a normalised liver architecture with less collagen structures after MSC or fibroblast administration (Fig. 6K).

**Figure 7:**
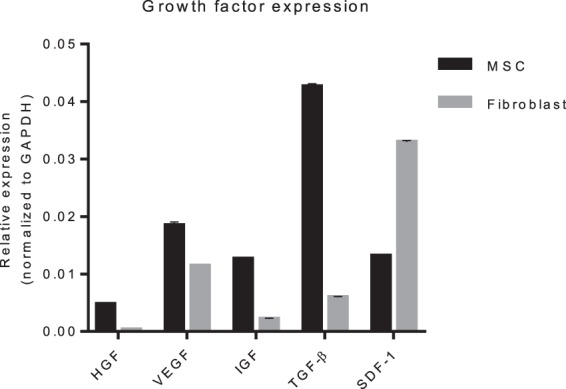
Pro-regenerative and fibrosis inhibitory gene expression levels in cultured MSCs and fibroblasts. Quantitative PCR for mRNA expression of pro-regenerative and fibrosis inhibitory genes in cultured MSCs and fibroblasts. Expression levels of HGF, VEGF, IGF-1, TGF-β and SDF-1 are normalized to GAPDH. The graphs represent values of two independent experiments (n = 2, ±SEM).

To evaluate how MSCs could potentially prevent progression of liver fibrosis, qPCR analysis on cultured cells was performed to determine if growth factors, which have been described to stimulate tissue regeneration and inhibit fibrogenesis, were expressed. Results showed that cultured MSCs express HGF, IGF-1, VEGF, TGFβ and SDF-1, all important factors in tissue regeneration and reversing fibrosis (Fig. 7). Fibroblasts had lower expression levels of these genes, except for SDF-1. Unfortunately analysis of the expression levels of these genes in the zebrafish embryo was not possible. The low number of cells injected resulted in expression levels below the detection limit of the qPCR.

Altogether these results indicate that MSCs ameliorate TAA-induced liver fibrosis in zebrafish embryos and show the applicability to use this model to test new therapeutic interventions for liver fibrosis.

## Discussion

Cirrhosis mostly evolves when the liver is chronically challenged by harmful impulses like alcohol or hepatitis viruses. Treatment is limited to the removal of the harmful stimuli or a liver transplantation^19,20^. Although many studies already have been done no real new therapeutic strategies to counter fibrogenesis have emerged yet. In most of these studies mice and rats were used as animal models which, are although valuable, are not suitable for high throughput screening. Our current study shows that zebrafish embryos can be used as a robust and reliable novel model system for liver fibrosis. We found that 0.06% TAA treatment induced liver fibrosis, characterised by increased RNA expression of collagen, Hand-2 and Acta-2, smaller liver sizes and collagen deposition. Applicability to analyse novel therapeutic interventions was shown by the administration MSCs and fibroblasts as potential novel cell therapies. Results showed that MSCs, in contrast to fibroblasts, were able to considerably prevent the progression of TAA-induced liver fibrosis in the zebrafish embryos.

Chronic intraperitoneal administration of CCL4 is a frequently used model to induce liver fibrosis in mice and rats. Due to the long time to induce fibrosis, the costs and workload these models are not suitable for high throughput screening^7,8^. Based on the results from the present study, CCL4 is not capable of inducing fibrosis in zebrafish embryos in non-toxic dosages. No structures of collagen deposition or upregulation of fibrotic, inflammation or damage related genes were observed in CCL4 injected embryos. Altogether this indicates that CCL4 yolk sac injections do not lead to liver fibrosis in zebrafish embryos.

Next, we studied the ability of different concentrations of TAA dissolved in egg water to induce liver fibrosis. Survival and liver size measurements reveal a toxic effect to the embryos since the two highest dosages of TAA (0.24% and 0.48%) were lethal and showed oedema in the heart cavity. Lower dosages of TAA were not lethal and led to smaller liver sizes after 6 days of treatment. The shrinkage of the liver is a well-known feature of fibrotic livers in animals and humans. Amali and Huang *et al*., like us, also observed this liver shrinkage when zebrafish embryos were treated with TAA or ethanol, respectively^12,16^. However, the TAA study of Amali *et al*. focused on a model for steatosis but did not evaluate the induction of fibrosis. In the present study we focused on the ability and mechanisms of TAA to induce fibrogenesis.

Sirius-red staining, showed structures of collagen deposition in the livers of the 0.03, 0.06 and 0.12% TAA treatment groups but looked different compared to the structures observed in fibrotic mouse and human livers^11^. This difference can be due to the well-known different cell organisation within the liver of the fish compared to humans. The zebrafish liver does not have the typical lobular architecture of the liver of human and mice consisting of 6 portal triads (location of stellate cells) surrounding the hepatocytes and one central vein in the middle. These veins, arteries, bile ducts, hepatocytes and stellate cells are less well organised in the zebrafish^7^. These fundamental architectural differences could be the reason that the collagen structures found upon TAA treatment looked different compared to the septa-like structures found in fibrotic livers of human and mice.

Our data show that collagen RNA expression was also upregulated in 0.06% and 0.12% TAA treated embryos. These results are in line with several other studies with mouse and adult zebrafish which also show increased expression of collagen RNA levels^11,13^. Surprisingly the fibrotic (Hand-2, Acta-2) and inflammatory/damage (TGF-β, SDF-1a and SDF-1b) related genes were only upregulated in the 0.06% TAA treatment group. We can speculate that the 0.12% TAA treatment was too toxic and induced another type of damage next to the induction of fibrosis. The increased expression levels of Hand-2 and Acta-2 (fish homologue of α-smooth muscle actin) indicates stellate cell proliferation, activation and, differentiation to myofibroblasts, which are responsible for the secretion of collagen^7,13–15^. This is a well-known and highly preserved mechanism of fibrogenesis in human and rodents and starts by the apoptosis of hepatocytes which initiates the activation of stellate cells. Research of Howarth *et al*. and other studies also observed increased expression levels of these fibrosis-related genes in adult fish livers upon ethanol treatment^15^. These data indicate that the mechanism of fibrogenesis in zebrafish embryos is highly similar to that in mice, rats and humans. Altogether these data showed that 6 day treatment of zebrafish embryos with 0.06% TAA induces liver fibrosis. The low labour intensiveness, cushiness and low costs of this TAA based model make it suitable for high throughput drug screening.

In the second part of our study we tested the applicability of this novel TAA model to screen for new therapeutics by testing the abilities of MSCs and fibroblasts to modulate the fibrotic process. Sakaida *et al*. published one of the first articles describing reduced CCL4 induced liver fibrosis in mice upon MSC treatment^33^. Over time more data were published on reduced liver damage with MSC treatment^25,29,30,32,34,35^. Until the present study, zebrafish embryos were never used to test the applicability of MSCs as treatment or preventive therapy for liver fibrosis. Our results show that local MSC treatment in our novel zebrafish embryo model can prevent the progression of fibrosis since after administration no collagen structures in the liver were observed and all the fibrotic-, inflammatory/damage- and liver function related- genes normalised. In addition, fibroblast and MSC treatment also abolished the TAA induced shrinkage of the liver. This could be due to the secreted growth factors like hepatocyte growth factor (HGF) and the survival stimulating factors of the MSCs on hepatocytes^24,35^. Administration of fibroblasts showed less modulation of this fibrotic process than with MSCs.

MSCs are known to play a role in cell survival, tissue regeneration and immune suppression^23,28^. Immune suppression cannot be the main working mechanism in the amelioration of fibrosis in zebrafish embryos since the T-cells are generated at 8dpf and are thus not present during the experimental period^36–38^. It could be that MSCs inhibit the proliferation of stellate cells indicated by the down-regulation of Hand-2 RNA levels. This event is also described by Najimi *et al*. who observed this MSC guided inhibition of stellate cell proliferation in mice^24,25^. In addition, it could be that MSCs prevent stellate cell activation or silence the activated stellate cells indicated by the downregulation of Acta-2 RNA levels. Previous studies suggest that there is a reduction in proliferation and silencing of myofibroblasts due to cytokines (IL-10, HGF, VEGF and IGF-1) secreted my MSCs leading to less ECM production in the liver^23,25,30,35,39^.

In line with these studies we showed that MSCs in culture express HGF, IGF, VEGF and TGFβ, which are all important in tissue regeneration and are also described to be important in reversing fibrosis. Fibroblasts had lower expression levels of these cytokines providing a potential explanation for the observed specific effect of MSCs, although additional studies would be required to explore this further.

Xenogenic or xenograft models in general always have some limitations since there are differences between species, which theoretically could lead to different experimental outcomes or hamper translation to humans^40,41^. This could also be the case for the present novel zebrafish model where we evaluated cellular therapeutic interventions to prevent progression of fibrosis. To illustrate its applicability we used mouse MSCs as proof of principle. Previous studies have shown that both mouse and human MSCs can reduce fibrosis in mouse models for liver fibrosis^24,25,29,31,32,35,39^. We think that the preserved pathogenesis of liver fibrosis in the zebrafish embryos (e.g., stellate cell activation, collagen deposition), we observed and its applicability to be used as a test model using mouse MSCs, makes it a valuable model for high throughput testing novel preventive or therapeutic interventions for liver fibrosis. Of course further investigations are required to validate this model and explore other applications.

Altogether our results indicate that mouse MSCs seem to have the same effect in zebrafish embryos and could also have the same working mechanism on liver fibrosis as observed in mouse models. This suggests the usefulness of this novel TAA induced zebrafish fibrosis model for drug screening.

In conclusion, TAA can induce liver fibrosis in zebrafish embryos. This probably acts through mechanisms similar to man and mice, thereby providing a promising and rapid model for future mechanistic and therapeutic studies on liver fibrosis, like we showed by the administration of MSCs. We showed that MSCs seem to prevent progression of liver fibrogenesis in this novel zebrafish embryo model. Therefore MSCs may be a promising novel therapy for patients with increased liver fibrogenesis.

## Material and Methods

### Induction of liver fibrosis in zebrafish embryos

Housing and experiments were done according to the Dutch guidelines for the care and use of laboratory animals and approved by the animal welfare committee of the Leiden University. Carbon tetrachloride (CCL4) and thioacetamide (TAA) were used to induce liver fibrosis in liver-fatty-acid-binding-protein (LFABP)-GFP zebrafish embryos from the Leiden University breeding facility, which only express GFP in the liver^42^. For the CCL4 model, zebrafish embryos were injected in the yolk sack once [2 days post fertilization (dpf)] or twice (2dpf and 4dpf) with 0.25 M CCL4 diluted in mineral oil or mineral oil control (Sigma-Aldrich Chemie BV, Zwijndrecht, The Netherlands) (Supplemental Fig. 1A,B). Different volumes (1 nl, 5 nl and 10 nl) of 0.25 M CCL4 were evaluated. Injections were done on tricaine mesylate (Sigma-Aldrich) tranquilized embryos, fixed to an agar coated plate with use of a microinjector (20 psi, PV820 Pneumatic PicoPump, World Precision Instruments) using needles pulled from borosilicate capillaries (O.D. 1.0 mm × I.D. 0.78 mm, Science products, Hofheim, Germany) and Leica MS55 stereo microscope visualization^43^.

For the TAA model, 2dpf zebrafish embryos were treated by adding TAA for 6 days in the egg water (water with 60 µg/ml instant ocean, sea salt). Different concentrations of TAA were tested (0.0075%, 0.015%, 0.03%, 0.06%, 0.12%, 0.24%) to evaluate the induction of fibrosis (Supplemental Fig. 1C).

At the end of the experiment, the embryos were imaged, fixated in 4% buffered paraformaldehyde for paraffin embedding or stored in PAXgene blood RNA solution (PreAnalytiX, Hombrechtikon, Switzerland) for RNA isolation. Furthermore, embryos were imaged by bright field and GFP fluorescent microscopy (2x magnification, Olympus IX53) to image the total embryo and the liver, respectively. Embryo and liver size were quantified using ImageJ analysis software (ImageJ 1.47v, National Institutes of Health, USA).

### MSC and fibroblast cell culture and treatment

MSCs were isolated from the bone marrow of 8–10 week old male mT/mG C57Bl/6Jico mice obtained from an LUMC breeding population. These mice express red fluorescence protein (RFP) in all cells^44^. Mice were sacrificed by cervical dislocation, femur and tibia were collected. Subsequently, bones were flushed with RPMI cell culture medium supplemented with 10% fetal calf serum (FCS; Gibco, 3mM L-glutamine (Invitrogen Corp., Paisley, UK), penicillin/streptomycin (Pen/Strep; Invitrogen Corp., Paisley, UK) and 2% Heparin (Pharmacy AZL). Isolated cells were cultured in αMEM culture medium (Lonza) supplemented with 10% FCS, 3mM L-glutamine and P/S. Non-adhering cells were removed by refreshing the media after 24, 48 and 72 h. GFP expressing colon fibroblasts were isolated from GFP-actin male mice and cultured in DMEM/F12 culture media supplemented with Pen/Strep and 10% FCS.

Approximately 100 MSCs or fibroblasts in 1 nl polyvinylpyrrolidone (PVP) or PVP only as a control were injected in closest proximity to the liver at 5dpf of 0.06% TAA treated or healthy zebrafish embryos. After cell therapy, TAA treatment was continued until the end of the experiment (Supplemental Fig. 1D). At the end of the experiment, the embryos were imaged and subsequently fixated in 4% paraformaldehyde for paraffin embedding and stored in PAXgene RNA storage solution for RNA isolation.

### Phenotypical and functional characterization of MSCs

To characterize the bone marrow derived MSCs, cells were incubated with fluorescent conjugated antibodies: CD29-PE-Cy7, C45-PE-Cy-7, SCA-1-PE-Cy-7, CD31-APC (eBioscience, Vienna, Austria), CD44-APC, CD105-BV786 or CD106-V450 (BD Pharmingen, San Diego, CA, USA). LSR II flow cytometer (BD Biosciences, San Diego, CA, USA), with FACS-diva software (version 8.7.1, Tree Star Inc. Ashland, OR, USA) were used to measure the fluorescence intensity. FlowJow software (version 8.7.1., Tree Star Inc. Ashland, OR, USA) was used for data analysis. Functional characterization was done by testing the ability of MSCs to differentiate in osteoblasts and adipocytes. Experiments were performed as earlier described by our group^45^. In short, MSCs were cultured with osteogenic or adipogenic differentiation medium. Osteogenic differentiation medium consists of complete medium with 10 nM dexamethason, 50 μg/ml ascorbic acid and 10 mM β-glycerophosphate (all from Sigma-Aldrich Chemie BV, Zwijndrecht, The Netherlands). Adipogenic differentiation medium consists of complete medium with 1 µM dexamethason, 5 µM insulin, 100 μM indomethacin and 0.5 mM 3-isobutyl-1-methylxanthine (all from Sigma-Aldrich Chemie BV, Zwijndrecht, The Netherlands). After 21 days, differentiation was verified by fast blue staining for alkaline phosphatase expression and Alizarin red for calcium deposition (both Sigma-Aldrich Chemie BV, Zwijndrecht, The Netherlands). Oil-red-O staining was used to visualize lipid droplets and used as a marker for adipogenic differentiation.

### Histological examination

Paraffin sections of 4 µm were cut, hydrated and stained with Sirius-red and Hematoxylin-Eosin (H&E) solutions. For Sirius-red staining, sections were incubated for 90 min with 1 g/L Sirius-red F3B in picric acid (both Klinipath) and subsequently incubated for 10 min with 0.01 M HCl. H&E staining was performed by 5 min incubation with Hematoxylin solution (Mayer, Merck) followed by a 10 min wash with tap water and a 30 seconds Eosin staining (Sigma). After the staining slides were dehydrated and mounted with Entellan (Merck KGaA).

### Immunohistochemistry

In order to detect the fibroblasts and MSCs in the zebrafish embryos, sections were stained for mouse specific Vimentin. In short, sections were hydrated and endogenous peroxidases were blocked by 20 min incubation with 0.3% H_2_O_2_/methanol at room temperature (RT). Next, antigen retrieval was performed by boiling the section for 10 min in citrate buffer (0.1 M, pH6.0), cooled down and incubated overnight with a rabbit anti mouse Vimentin antibody at 4 °C (Cell signalling). Subsequently, slides were incubated for 1 h with a secondary goat anti rabbit-HRP conjugated antibody followed by a 10 min incubation with 3,3′-diaminobenzidine (DAB Fast Tablet, Sigma-Aldrich, St. Louis, MO). Nuclear counterstaining was performed with Hematoxylin after which the sections were dehydrated and mounted with Entellan.

### RNA isolation, cDNA synthesis and Quantitative polymerase chain reaction (qPCR)

Zebrafish embryos (N = 25) were dissolved for 2 days at 4 °C in PAXgene Blood RNA solution (PreAnalytiX). mRNA from the total embryo lysates, MSCs and fibroblasts was isolated with NucleoSpin RNA kit (Machery-Nagel GmbH, Düren, Germany). cDNA was synthesised with 1 μg RNA incubated with M-MLV transcriptase, dNTPS, random primers and RNasin ribonuclease inhibitor according to manufecturers’ protocol (Promega, Madison, Wisconsin, USA). Quantative PCR was performed with a mix consisting of 1 nl cDNA, 1 nM primer and 5 μl iQ SYBR Green supermix reagent (Bio-Rad Laboratories, Berkeley, California, USA). In mRNA samples of the zebrafish embryos, expression levels of fibrosis- (Colagen-1α1, Hand-2, Acta-2), inflammatory- (TGF-β, SDF-1a, SDF-1b) and liver function genes (group-specific component, alpha-1 antitrypsin and serum amyloid A) were evaluated and normalised to ribosomal protection protein (RPP), which was used as reference gene. mRNA expression of pro-regenerative and fibrogenesis inhibitory genes (HGF, VEGF, IGF-1, TGF-β and SDF-1) in the cultured MSCs and fibroblasts levels was measured and normalised to GAPDH, which was used as reference gene (Supplemental Table 1: primer sequences).

### Statistical Analysis

GraphPad Prism software was used for statistical analysis and p-values lower than 0.05 were considered to be statistically significant (GraphPad Software, version 5.01, San Diego, CA). One-Way ANOVA test was used to compare 3 or more groups. Two groups comparisons were performed with Student’s t-test. Results are displayed as the means ± standard error of the mean (SEM). All data shown are from two or three independent experiments, using group sizes of 25–50 embryos.

## Electronic supplementary material

Supplemental information
