# Supplementary material for: Mesenchymal stromal cells prevent progression of liver fibrosis in a novel zebrafish embryo model
Source: Sci Rep. 2018 Oct 30;8:16005. doi: 10.1038/s41598-018-34351-5 (PMC6207680; doi:10.1038/s41598-018-34351-5)
Supplement: Supplementary file 1 — Supplemental information [file 41598_2018_34351_MOESM1_ESM.pdf]

Mesenchymal stromal cells prevent progression of liver fibrosis in a novel zebrafish embryo model

Supplemental Information

Danny van der Helm[1], Arwin Groenewoud[2], Eveline S.M. de Jonge-Muller[1], Marieke. C. Barnhoorn[1], Mark J.A. Schoonderwoerd[1], Minneke J. Coenraad[1], Lukas J.A.C. Hawinkels[1], B.Ewa Snaar-Jagalska[2], Bart van Hoek[1 +], Hein Verspaget[1 +,*]

[+] joint senior authorship

[1]Department of Gastroenterology and Hepatology, Leiden University Medical Center, Leiden, the Netherlands.

[2]Department of Animal Science and Health, Leiden University, Leiden, the Netherlands.

[*]Corresponding Author:

Prof. dr. H. Verspaget MSc, PhD

Department of Gastroenterology and Hepatology

Leiden University Medical Center

P.O. Box 9600, 2300 RC Leiden

The Netherlands

Telephone/Fax: +31 71 526 2680/+31 71 524 8115

E-mail: H.W.Verspaget@lumc.nl

Supplemental table 1: Primer sequences

Gene	Abbreviation	Forward	Reverse
**Zebrafish**			
α1-antitrypsine	α1AT	CATGTTGGGTCACAGTCAGG	CGATTTCAGGCTTGGAGAA
ACTA-2	ACTA-2	TTGTGCTGGACTCTGGTGAT	GGCCAAGTCCAAACGCATAA
Collagen-1α1	Col-1α1	CTTTTGCTCACAGGGCCTTT	AAGACTGCATGCATCACAGC
Vitamin D-binding protein	GC	ACTCTCCATTCCCCAAGCAT	TAGCGAAGTGAAGCCAGACA
HAND-2	HAND-2	CCTTCAAAGCGGAATTCAAA	CAGATGGCCTCATTTCGTCT
Ribosomal Protection protein	RPP	CTGAACATCTCGCCCTTCTC	TAGCCGATCTGCAGACACAC
Serum amyloid A	SAA	CGTGCCTACCAGCATATGAA	CAGCATCTGAATTGCCTCTG
Stromal derived factor 1a	SDF-1a	CGCCATTCATGCACCGATTTC	GGTGGGCTGTCAGATTTCCTTGTC
Stromal derived factor -1b	SDF-1B	CGCCTTCTGGAGCCCAGAGA	AGAGATTCTCCGCTGTCCTCC
Transforming growth factor-β	TGF-β	CCTTGCTTGCTGGACAGTTT	AATCCGCTTCTTCCTCACCA
**Mouse**			
Hepatocyte growth factor	HGF	AAGAGTGGCATCAAATGCCAG	CTGGATTGCTTGTGAAACACC
Vascular endothelial growth factor	VEGF	CACAGCAGATGTGAATGCAG	TTTACACGTCTGCGGATCTT
Insulin-like growth factor	IGF	CTACAAAAGCAGCCCGCTCT	CTTCTGAGTCTTGGGCATGTCA
Transforming growth factor-β	TGF-β	CAACAATTCCTGGCGTTACC	TGCTGTCACAAGAGCAGTGA
Stromal derived factor 1	SDF-1	GAAAGGAAGGAGGGTGGCAG	TCCCCGTCTTTCTCGAGTGT
Glyceraldehyde 3-phosphate dehydrogenase	GAPDH	AACTTTGGCATTGTGGAAGG	ACACATTGGGGGTAGGAACA

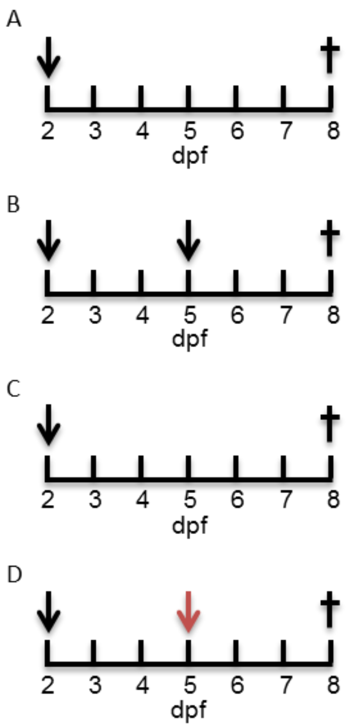

Supplemental Figure 1. Schematically treatment scheme for CCL4, TAA and cell administration.

CCL4 was one (A) or two (B) times injected in the yolk sac of zebrafish embryos (black arrows). Embryos were sacrificed 8dpf (cross). (C) TAA was dissolved in the egg water of 2dpf zebrafish embryos (black arrow). Embryos were kept in this water until sacrifice 8dpf (cross). (D) TAA treated (black arrow) or control zebrafish were injected at 5dpf (red arrow) with MSCs, fibroblasts or PVP. Embryos were sacrificed 8dpf (cross).

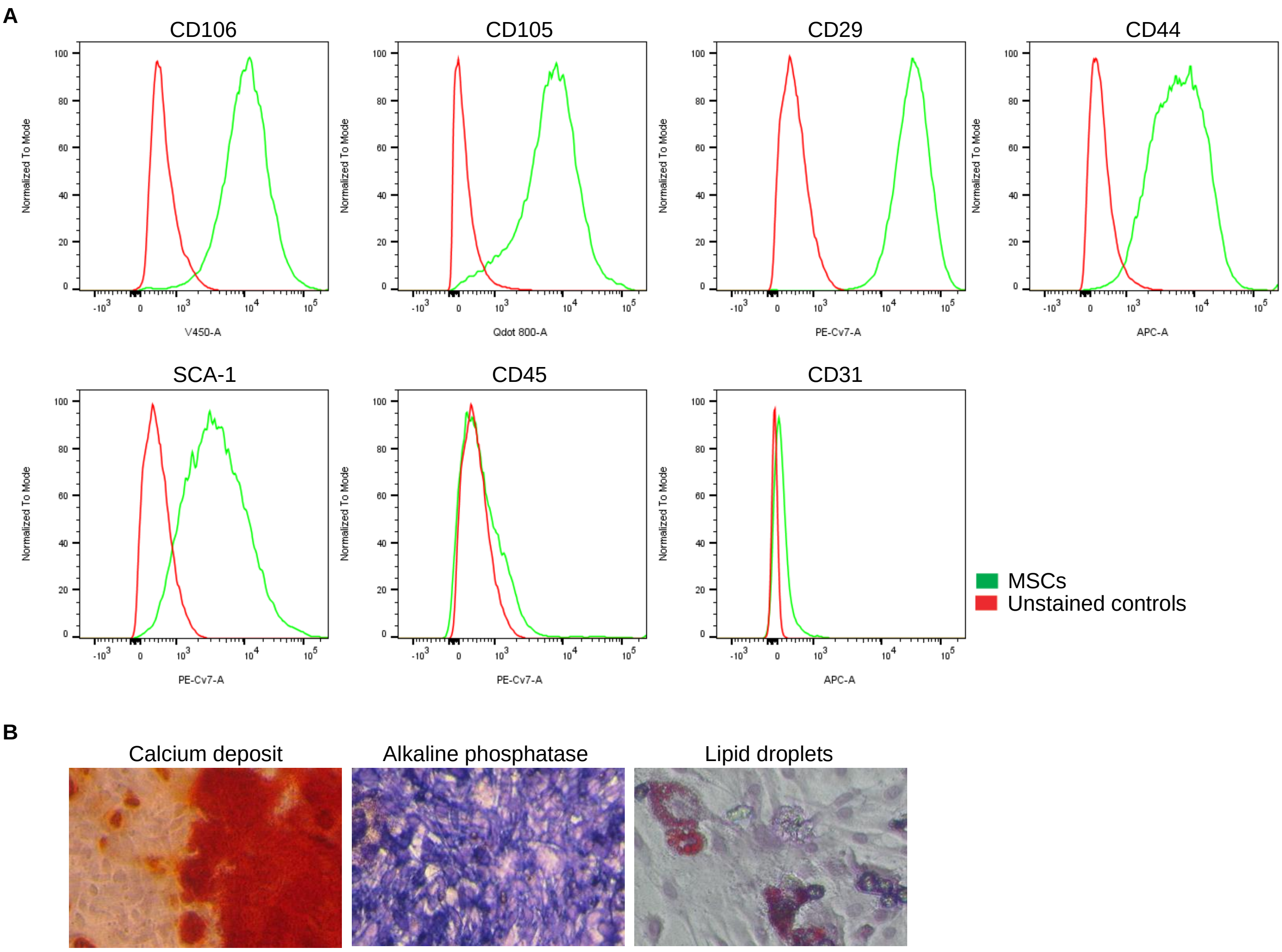

Supplemental Figure 2. Phenotypical and functional characterization of MSCs

MSCs were isolated from bone marrow of 8-10 week old mT/mG C57Bl/6Jico mice. MSCs were characterised by membrane markers and osteoblast and adipocyte differentiation. (A) CD106, CD105, CD29, CD44, SCA-1, CD45 and CD31 membrane markers were measured by flow cytometry. (B) Osteoblast differentiation was visualised by calcium deposit (Alizarin red staining) and upregulation of alkaline phosphatase (fast blue staining). Adipocyte differentiation was visualised by cytoplasmic lipid droplets (oil-red-o) staining.
